# Magnetic structure determination of multiple phases in the multiferroic candidate GdCrO_3_

**DOI:** 10.1107/S2052520625001921

**Published:** 2025-04-14

**Authors:** Pascal Manuel, Dmitry Khalyavin, Fabio Orlandi, Laurent Chapon, Wang Xueyun, Tae-Hwan Jang, Eun Sang Choi, Sang-Wook Cheong

**Affiliations:** a ISIS Neutron and Muon Source Rutherford Appleton Laboratory DidcotOX11 0QX OxfordshireUK; b Diamond Light Source Ltd Harwell Science and Innovation Campus Didcot OxfordshireOX11 0DE UK; c Keck Center for Quantum Magnetism and Department of Physics and Astronomy Rutgers, The State University of New Jersey 136 Frelinghuysen Road Piscataway NJ088548019 USA; d School of Aerospace Engineering Beijing Institute of Technology Beijing100081 People’s Republic of China; e MPPHC-CPM, Max Planck POSTECH/Korea Research Initiative, Pohang37673, Republic of Korea; f National High Magnetic Field Laboratory Florida State University Tallahassee FL32310-3706 USA; Oak Ridge National Laboratory, USA

**Keywords:** magnetic structure, perovskite, multiferroicity, neutron diffraction, spin re-orientation, incommensurate magnetic structure

## Abstract

Due to their potential applications in low-power consumption and/or multistate memory devices, multiferroic materials have attracted a lot of attention in the condensed matter community. As part of the effort to identify new multiferroic compounds, perovskite-based GdCrO_3_ was studied in both bulk and thin film samples. A strong enhancement of the capacitance in a field suggested ferroelectric behaviour but significant leakage and no well developed *P*–*E* hysteresis loops were observed. Measurements clearly indicate the existence of a polar phase but only below 2 K (likely connected to Gd ordering). Here the determination of the magnetic structure through neutron diffraction collected on an isotopic ^160^GdCrO_3_ sample at the WISH diffractometer at ISIS is reported. The presence of three successive magnetic phases as a function of temperature (commensurate, spin re-orientation and incommensurate phases once the Gd order), previously only seen by magnetization, is confirmed. Using the most recent guidelines for reporting the determined structures, we highlight the benefits of using such nomenclature for discussing physical properties and consider possible mechanisms and couplings that led this seemingly rather isotropic system to display the complex structures observed.

## Abstract 2

Using neutron powder diffraction, the successive magnetic structures in multiferroic candidate GdCrO_3_ have been determined, including a complex incommensurate phase below 2.3 K. The symmetry analysis uses a combination of little group formalism and superspace group approach highlighting the strength of each method and the results are reported using the latest IUCr guidelines.

## 1. Introduction

Multiferroic (MF) materials, exhibiting two or three of the properties ferroelasticity, ferromagnetism/antiferromagnetism and ferroelectricity/antiferroelectricity, have generated a huge interest in recent years (Kimura *et al.*, 2003 ▸; Spaldin & Fiebig, 2005 ▸) due to potential applications in new types of memory devices, actuators and transducers. The most common family of MF materials are perovskite-based phases *AB*O_3_. Aristotype perovskites can be described by a cubic lattice, where the usually larger *A* cations sit at the centres and the *B* cations at the corners, in an octahedral oxygen coordination. In the distorted perovskite structures, the off-centering of the cations is responsible for the ferroelectricity. One strategy for making MF materials is to substitute one (or both) of the sites with magnetic ions. GdCrO_3_ is a distorted perovskite which crystallizes in space group *Pbnm*.1′ (No. 62) with *a* ∼  5.31 Å, *b* ∼ 5.51 Å and *c* ∼ 7.61 Å (Geller, 1957 ▸) or equivalently *Pnma*.1′ with *a* ∼ 5.51 Å, *b* ∼ 7.61 Å, *c* ∼ 5.31 Å. Whilst the literature on orthoferrites and orthochromates tends to use this non-standard notation, we have chosen to use the standard crystallographic notation *Pnma*.1′ for reporting crystallographic information throughout this paper but provide the conversion to *Pbnm*.1′ setting when appropriate. We will also use the Belov–Neronova–Smirnova (Belov *et al.*, 1957 ▸) notation (BNS) to indicate the magnetic space groups (MSG). A comprehensive study of GdCrO_3_ magnetic properties dates from 1974 (Cooke *et al.*, 1974 ▸); since then, no attempt has been made to study its low-temperature magnetic structure. It was later reported that GdCrO_3_ could be another perovskite-based MF material in both bulk and thin film samples grown on Pt/Si substrate (Cheng *et al.*, 2010 ▸). However, the authors themselves admit the evidence for polarization is ambiguous. This is reminiscent of biferroic behaviour recently observed in YCrO_3_ (Serrao *et al.*, 2005 ▸).

**Figure 1:**
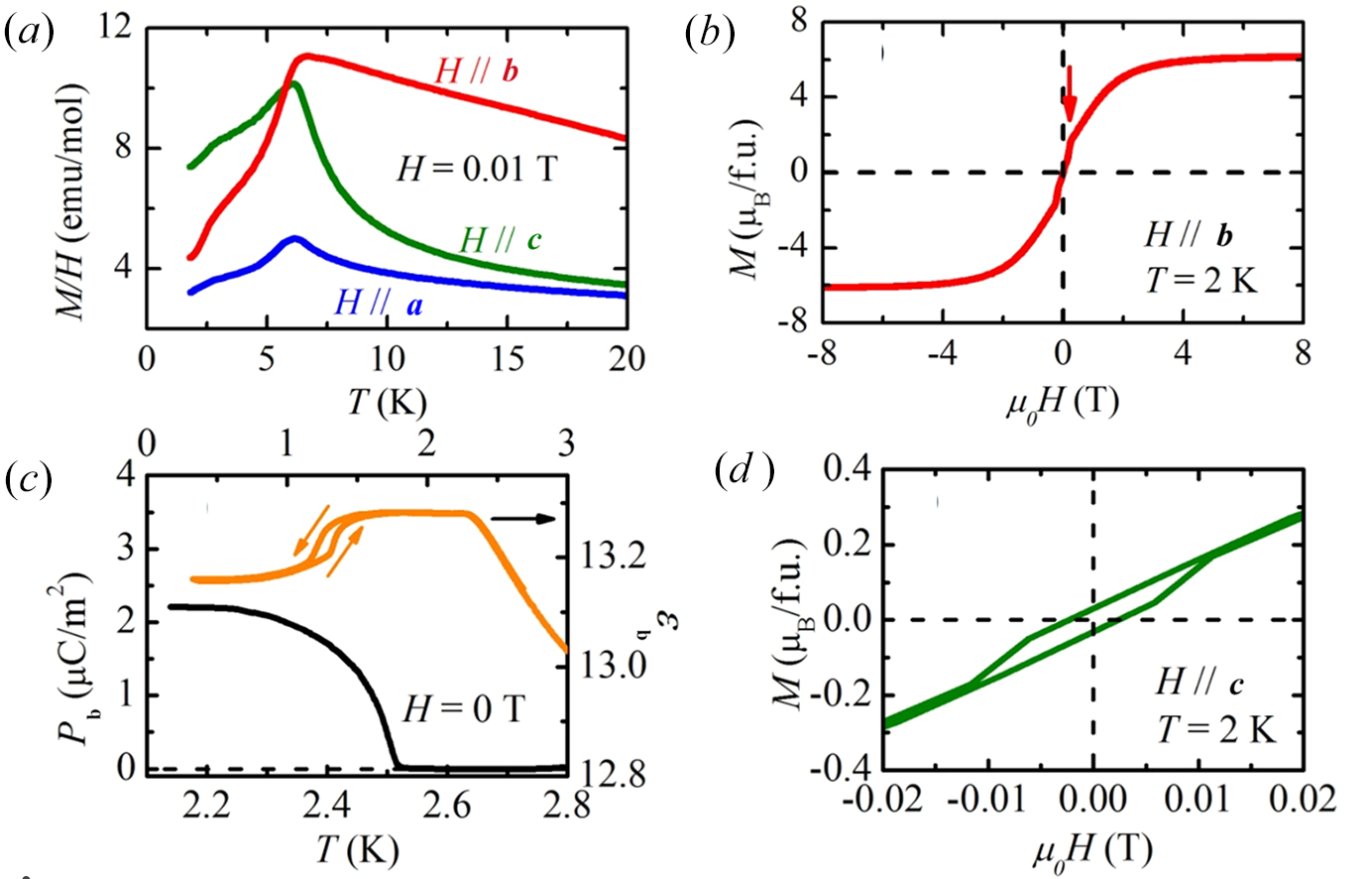
(*a*) Susceptibility as a function of temperature near the spin re-orientation region along the three orthogonal directions of the GdCrO_3_ crystal and (*b*) magnetisation as a function of field applied along the *b* axis showing a metamagnetic transition at *T* = 2 K. (*c*) Polarisation and permittivity as a function of temperature and (*d*) magnetisation as a function of field applied along the *c* axis at *T* = 2 K, showing the presence of a small hysteresis.

In contrast, we present clear evidence for the existence of a polar phase below 2.3 K, see Fig. 1 ▸, with polarization developing below 2.3 K. Most importantly, we present new high-resolution neutron diffraction data measured at 300 K to 1.5 K; thus, providing a new insight into the magnetic behaviour of GdCrO_3_. We re-examine the spin reorientation below 8 K, bringing into light new information compared to a recent hot neutron study (Tripathi *et al.*, 2019 ▸), and report a new incommensurate phase below 2.3 K.

## 2. Macroscopic properties

High-quality polycrystalline GdCrO_3_ was prepared using a solid-state reaction method. Gd_2_O_3_ with ^160^Gd isotope (98%+ purity purchased from CK Gas Product Ltd, UK) and Cr_2_O_3_ were mixed thoroughly, then pelletized. The pellet was heated at 1473 K for 12 h. After first sintering, the polycrystalline sample was re-ground and pelletized, then sintered at 1523 K for 24 h. The last sintering temperature was 1573 K for 30 h. Single crystals of GdCrO_3_ were grown using a flux method (Wanklyn, 1969 ▸). A mixture consisting of 15 g of Gd_2_O_3_, 6.2 g of Cr_2_O_3_, 107 g of PbF_2_, 1 g of PbO, and 3 g of B_2_O_3_ was carefully placed in a 100 cm^3^ platinum crucible with a closely fitting lid. This mixture was then heated to a temperature of 1553 K and maintained at this level for 5 h to ensure complete dissolution of the components in the flux. Following this, the mixture was slowly cooled at a rate of 1.5 K per hour down to 1173 K, allowing for the gradual crystallization of the desired material. Once cooled, the as-grown crystals were mechanically separated from the crucible. The flux was easily removed using diluted nitric acid, resulting in clean crystals with sizes ranging from 2 to 3 mm. Single crystalline specimens were oriented along crystallographic directions using back scattering Laue diffraction. Magnetoelectric properties on a GdCrO_3_ crystal were obtained on a physical property measurement system from Quantum Design. Temperature (*T*) and magnetic field (*H*) dependence of dc magnetization was studied using vibrating sample magnetometry. Thin plate-shaped specimens were prepared for measurements of dielectric permittivity (ɛ) and electric polarization (*P*) along the unique axis. *T*-dependent *P*_c_ was obtained by integrating pyroelectric currents from an electrometer (Keithley-6517A). After cooling the specimen from 10 K to 2 K under *E* = 2.4 MV m^−1^, pyroelectric current was measured during the heating process at 4–5 K min^−1^ of the *T* sweep rate. ɛ_c_ below 3 K was collected at National High Magnetic Field Laboratory (Tallahassee, USA) using a capacitance bridge (AH-2700 A) under an excitation voltage of 1 V and an external frequency of 10 kHz.

From magnetization versus field data on single crystal and heat capacity data, Cooke *et al.* (1974 ▸) identified three magnetic transitions: Cr ordering at 170 K, a spin reorientation at ∼7 K and finally Gd ordering at 2.3 K. The successive transitions can be understood in terms of the relative strength of the interactions: at first, only Cr–Cr interactions are relevant, upon cooling Cr–Gd interactions become important and finally Gd–Gd start to play a role. Our own magnetization measurements confirm the existence of these transitions and, focusing on the behaviour below 20 K (Fig. 1 ▸), also reveal that the low-temperature phase (*T* < 2.3 K) displays weak ferromagnetism along *c* as well as a metamagnetic transition at low fields. No magnetic structure of this material, necessary to understand the mechanism behind its MF properties, has been reported thus far in this low-temperature phase (*T* < 2.3 K).

Cooke *et al.* (1974) ▸ rationalized the successive magnetic transitions by assuming the absence of distortion lowering the symmetry and drawing on the group theoretical consideration work of Bertaut (1963 ▸) and experimental work on other members of rare earth orthochromates (Mareschal & Sivardière, 1969 ▸) and predicted a *G*_*x*_F_*z*_ state below 170 K and a *G*_*z*_F_*x*_ state below 7 K but did not speculate on the structure below 2.3 K. Below 170 K, the canting of the Cr moments out of the collinear antiferromagnetic alignment produces a net magnetic moment and thereby an effective field at the Gd sites and a paramagnetic moment *M*_Gd_, whose direction is opposite to the canted Cr moment. Additionally, they attributed this canting to the Dzyaloshinskii–Moriya (DM) antisymmetric exchange interaction between the Cr^3+^ cations.

Recently, some ambiguous evidence (significant leakage and no well developed *P*–*E* hysteresis but strong enhancement of the capacitance in a field) for ferroelectricity at room temperature has been observed in both bulk and thin films (grown on Pt/Si substrate) samples of GdCrO_3_ (Cheng *et al.*, 2010 ▸). The unusual polarization data is similar to the biferroic behaviour observed in YCrO_3_ (Serrao *et al.*, 2005 ▸), which was later tentatively explained via local noncentrosymmetry observed through neutron pair distribution function (Ramenesha *et al.*, 2007 ▸). By contrast, our polarization measurements presented in Fig. 1 ▸ clearly reveal the existence of a polar phase below the 2.3 K transition. The permittivity measurements (Fig. 1 ▸) also pick up the 2.3 K transition and additionally exhibit another transition below 1.5 K with hysteretic behaviour. This ultra-low-temperature transition is ignored in the remainder of this paper as this is the only probe we used with *T* < 1.5 K.

A recent detailed single-crystal study (Yin *et al.*, 2015 ▸) revealed a giant magnetocaloric effect with an entropy change −Δ*S*_M_ at *T* = 3 K reaching about 29.5 and 31.6 J kg^−1^ K^−1^ for Δ*H* = 40 and 44 kOe, respectively, as well as confirming the occurrence of a spin re-orientation, negative magnetization, temperature-induced magnetization reversal and observing temperature-induced magnetization jumps in low fields.

## 3. Neutron diffraction

Neutron diffraction data were collected on the WISH diffractometer at ISIS Neutron and Muon Facility (UK) (Chapon *et al.*, 2011 ▸). Natural Gd has an extremely high-absorbing cross section for neutrons and, therefore, the ^160^Gd isotope was used to prepare the sample. The resulting powder was placed in a 3 mm-diameter vanadium can to minimize the presence of other isotopes, which, though present in small quantities, contributed to absorption which could still be seen in the data and included in the refinements. For all magnetic structures, mcif files (Perez-Mato *et al.*, 2015 ▸; https://www.cryst.ehu.es/ in particular *MVISUALIZE*) are given in supporting information.

### 3.1. High-temperature magnetic phase

Rietveld refinements in the paramagnetic phase confirm that GdCrO_3_ crystallizes in space group *Pnma*.1′, commonly observed for rare earth orthochromates and orthoferrites, with *a* = 5.522 (1) Å, *b* = 7.599 (2) Å, *c* = 5.309 (1) Å at 200 K. The Wyckoff positions, relevant for the magnetism, are Gd (4*c*) (0.06, ¼, −0.014) and Cr (4*b*) (0, 0, ½). Below 170 K, long-range magnetic order is observed with noticeably two strong peaks, growing in tandem as the temperature decreases, at 4.36 and 4.47 Å in *d*-spacing, corresponding to 011 and, the structurally forbidden, 110 Bragg peaks. Rietveld refinements show that this phase is characterized by a *G*_*z*_ ordering consistent with the mΓ_4_^+^ irreducible representation (irrep). It should be noted that *F*_*y*_ and *A*_*x*_ components are also allowed but not directly observed. Moreover, it is possible to add a contribution from mΓ_2_^+^ in the refinement but the improvement on 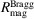 is marginal so it is due to the fact that both irreps bring a contribution to the same peaks in different proportions and therefore a combination of both with more refineable parameters will inevitably fit better. In contrast, forcing other irreps in isolation returns a significantly poorer 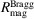 ∼20–30%, bringing confidence that mΓ_4_^+^ is the irrep chosen by the system in this high-temperature phase. The magnetic moments on each Cr atom refined at 20 K are provided in Table 1 ▸. Alternatively, we can describe this magnetic structure using the MSG *Pn*′*ma′* defined in the same unit cell and origin as the parent *Pnma* cell (details given in Table 2 ▸). An .mcif file containing the refined structure is provided as supporting information.

### 3.2. Spin reorientation

**Figure 2:**
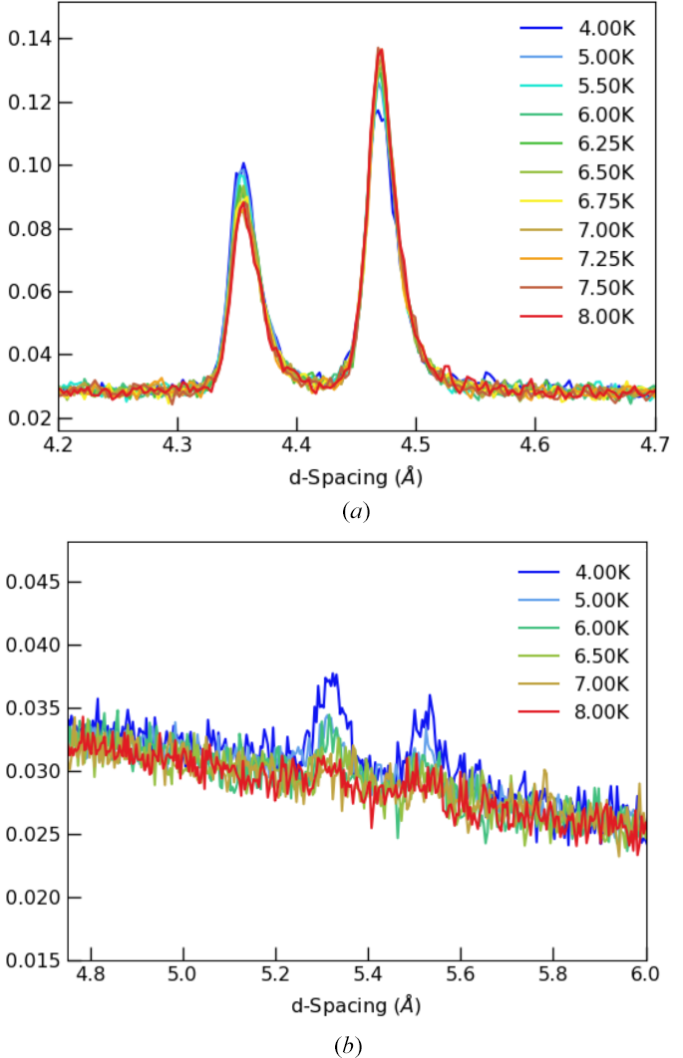
(*a*) Temperature evolution of the magnetic Bragg peaks 011 and 110 between 4 K and 8 K (90° detector bank). (*b*) Selection of the diffraction patterns (58° detector bank) showing the appearance of weak peaks (001 and 100) above 5 Å in *d*-spacing accompanying the spin reorientation.

Below ∼8 K, neutron diffraction reveals signs of the spin re-orientation consistent with earlier magnetometry measurements. A classic tell-tale sign of a spin re-orientation is the temperature dependence of the intensities of the 011 and 110 peaks, which shows an obvious intensity redistribution with the weaker peak growing whilst the stronger peak decreases as a function of temperature. This can be seen in Fig. 2 ▸. We used this intensity redistribution to estimate the temperature at which the system is experiencing a spin re-orientation: from 170 K to 20 K, both peaks grow as the temperature is lowered; cooling further, at 10 K, the intensity of both peaks does not change and by 8 K, the intensity redistribution is starting.

**Figure 3:**
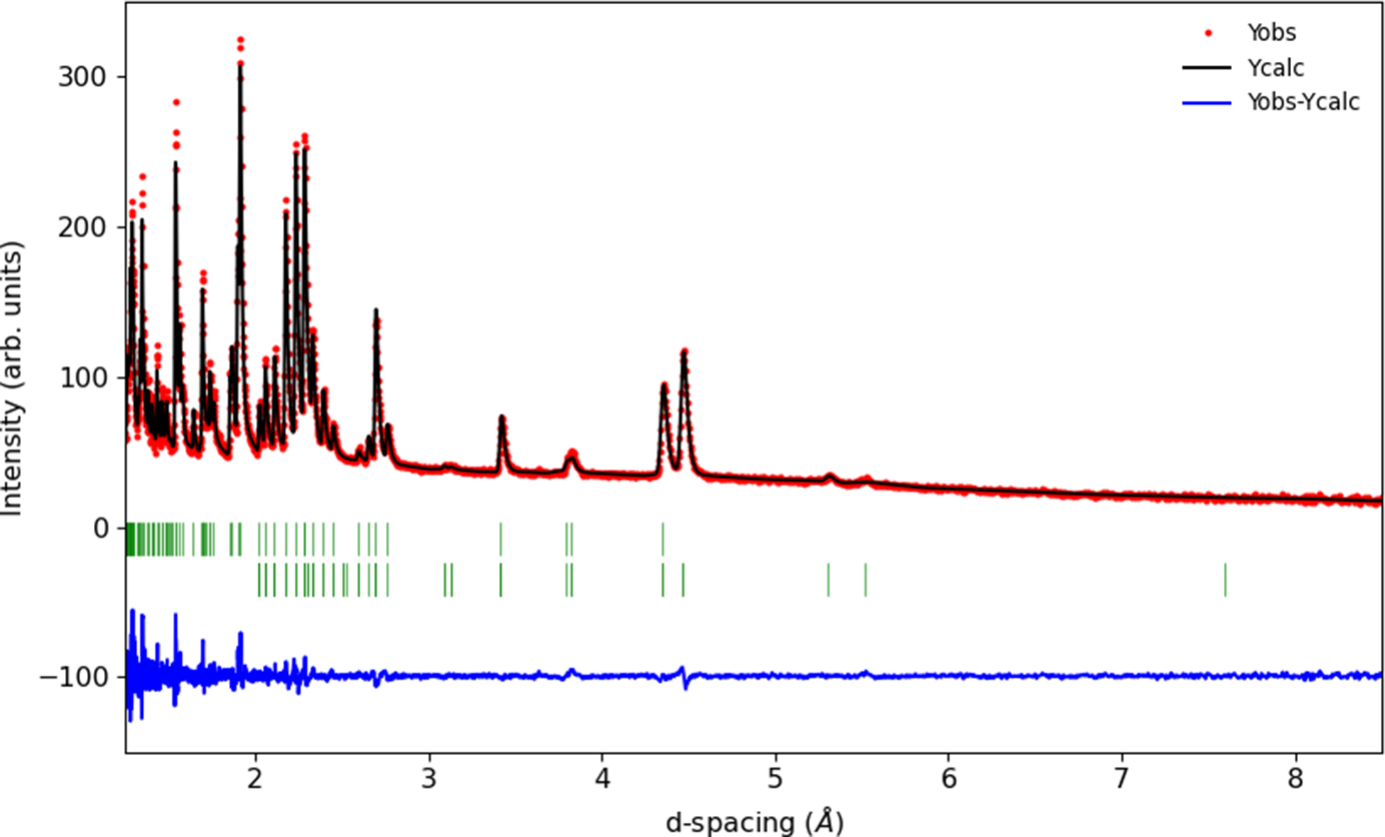
Rietveld refinement plot for GdCrO_3_ at 5 K showing the observed data (red points) as well as the calculated (black line) and difference (blue lines) profiles. The green tick marks represent the positions of the nuclear (top) and magnetic (bottom) reflections.

Assuming an irreducible magnetic-order parameter, the best fitting of the data collected at 5 K could be achieved using an mΓ_2_^+^ irrep, transforming as G-type spin ordering with the moment polarized along the *b* axis of space group *Pnma*.1′. The spin re-orientation is therefore predominantly G_*z*_(F_*y*_) to G_*y*_(F_*z*_). The high quality of our neutron data allowed the detection of two small peaks indexing as 001 and 100, see Fig. 2 ▸. It is impossible to account for the intensity of these small magnetic peaks without including a finite moment on the Gd sublattice. This is easily explained as the mΓ_2_^+^ irrep transforms two Gd modes, one of them is ferromagnetic along the *c* axis and the other is antiferromagnetic along the *a* axis. Attempts at refining the ferromagnetic component failed because the magnetic contribution resides on top of the strong nuclear Bragg peaks. However, the antiferromagnetic component is required to obtain satisfactory refinement quality. The refinement could be further improved if the Cr moments are allowed to deviate from the *b* axis within the *bc* plane (admixture of mΓ_4_^+^ irrep). This implies that the spin reorientation process is not completed at 5 K. The final refinement at 5 K is shown in Fig. 3 ▸ and the associated parameters are given in Table 1 ▸. The presence of the mΓ_2_^+^ modes at low temperature is fully consistent with magnetic structures predicted based on magnetization measurements (Belov *et al.*, 1957 ▸). A recent neutron study (Tripathi *et al.*, 2019 ▸) using natural Gd and a hot neutron diffractometer with λ = 0.4994 Å reported a better refinement at 15 K using a combination of mΓ_2_^+^ and mΓ_4_^+^. The data presented here have much higher resolution, clearly separating the contribution from the various peaks. We note that the previous study attributed moment components in all allowed directions for Cr and Gd moments, which is not required to obtain a good refinement in our data. From the temperature dependence of the moments, the authors argued that the negative magnetization is not driven by long-range order and used a neutron pair distribution function method to reveal significant antiferromagnetic correlations up to 9 Å. Additionally, Tripathi *et al.* (2019 ▸) report the appearance of 100, 010 and 001 (*Pbnm*) magnetic peaks. Our high quality and high-resolution data show that the latter (010 in *Pnma*) is not present. It is however very interesting that the presence of the 100 (*Pnma*) reflection is unambiguously confirmed by high-resolution diffraction. None of the Gd modes compatible with the two irreps used can account for this reflection. It can however be fitted if the secondary antiferromagnetic Gd-mode deviates from the *a* axis, implying an admixture of a third mΓ_1_^+^ irrep. Attempts to refine the three irrep model on the Cr sublattice failed due to the limited number of magnetic reflections and the parameters correlating too much. The two irrep model used in the present study (mΓ_2_^+^ + mΓ_4_^+^) results in the *P*2_1_′/*c*′ MSG described in the unit cell obtained using the following transformation: −*c*, −*a*, *b*+*c*; 0, 0, 0 (details given in Table 3 ▸). An .mcif file for this structure is provided in the supporting information.

### 3.3. Low-temperature phase at *T* = 1.5 K

**Figure 4:**
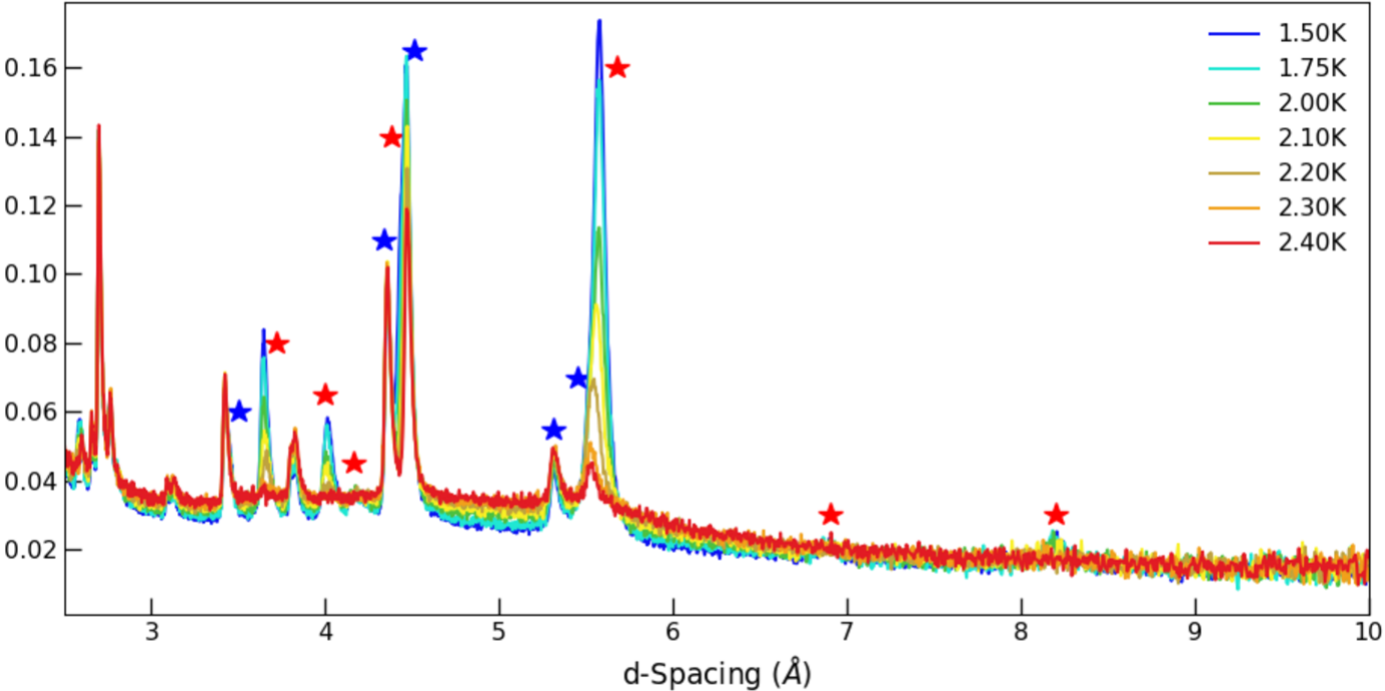
Temperature evolution of the neutron diffraction patterns between 1.5 K and 2.4 K, focusing on the long *d*-spacing region (58° detector bank) to highlight the magnetic peaks. **k** = 0 and incommensurate peaks are indicated by blue and red stars, respectively.

We now turn our attention to neutron data collected below 4 K. In accordance with the presence of another transition in the magnetometry, also seen in our polarization measurements (Fig. 1 ▸), there are significant changes to the neutron pattern below 2.3 K. A large number of new peaks appear and become very strong as the system is further cooled (Fig. 4 ▸). This resolutely indicates an incommensurate structure associated with Gd-sublattice ordering, although clearly a **k** = 0 component remains. These reflections can be indexed with an incommensurate propagation vector **k**_Gd_ = (*k*_*x*_, 0, *k*_*z*_), where *k*_*x*_ = 0.3294 (9) and *k*_*z*_ = 0.080 (2) at *T* = 1.5 K. We note that a recent study of the orthoferrite DyFeO_3_ (Ritter *et al.*, 2021 ▸) also revealed an incommensurate phase at low temperatures with **k**_Dy_ = (0, 0, 0.028) (in *Pbnm*.1′ setting) but that the propagation vector is distinctly different, being along a line of symmetry rather than within a plane of symmetry for GdCrO_3_.

To refine the incommensurate magnetic order of Gd, we first neglected the Cr sublattice and tested symmetry distinct models of the Gd sublattice. This approach allows us to constrain the refinement, and it is justified by the fact that both sublattices order independently with different propagation vectors. We used simple symmetry arguments based on the extended little group (Section 3.4), followed by a superspace group approach (Section 3.5) to show the advantages of using both formalisms. After establishing the Gd order, the full symmetry of the system was evaluated (including the Cr moments) and discussed in the context of the MF properties of GdCrO_3_.

### 3.4. Symmetry analysis: little group formalism

The extended little group of **k**_Gd_ (set of symmetry elements that do not change the propagation vector or transfer it to −**k**_Gd_) contains a screw axis along the *b* direction {2_010_|0,½,0}, inversion {−1|0,0,0} and the *ac* mirror plane {m_010_|0,½,0}, where we have used Seitz notation for the symmetry operations in the *Pnma*.1′ setting. This implies that the most symmetric variant of the magnetic structure has the nonpolar point group *2*/*m*.1′ (unique axis *b* of the parent *Pnma*.1′ structure). A symmetry element belongs to a magnetic point group if the corresponding transformation is equivalent to a phase shift of the modulation, which can then be compensated by an appropriate lattice translation multiplied by the propagation vector. For instance, in the simplest case of a single magnetic atom per unit cell, a spin density wave preserves inversion symmetry while cyclo­idal or helical structures do break it. The other two symmetry elements of the extended group can be preserved or broken by the cyclo­idal and helical ordering depending on the spatial orientation of the spin plane. If the plane is parallel to mirror or the mirror plane is perpendicular to the propagation vector, then the cycloid preserves these symmetry elements. If the mirror and the spin planes are perpendicular and the propagation vector is parallel to the mirror, then this symmetry element is lost. Helical structures cannot have any mirrors in their point group because they change helicity (handedness of the spin rotation upon propagation through the crystal). The situation is different for a twofold axis, it is preserved by helical and cyclo­idal structures, if the spin plane contains this axis and the axis is perpendicular to the propagation vector. However, if the spin plane of the cycloid is perpendicular to the twofold axis or the twofold axis is along the propagation vector then these symmetry elements do not belong to the magnetic point group. In contrast, a helical structure preserves the twofold axis which is parallel to its propagation vector.

If the unit cell contains more than one magnetic atom, the symmetry properties of the magnetic structure should be anticipated by considering what the appropriate symmetry element (interchanging the sites) does with the spin plane and the sense of spin rotation. The extended little group splits the Gd Wyckoff position 4*c* into two inequivalent sites, each with multiplicity two, Gd1_1 (−0.01540, 0.05888, 0.25), Gd1_2 (0.01540, −0.05888, 0.75) and Gd2_1 (0.48460, 0.44112, 0.75), Gd2_2 (0.51540, 0.55888, 0.25). The magnetic structure with point group 2/*m*.1′ has a spin-density-wave type of ordering, which is not an ideal candidate for the ground state of an insulating system with localized electrons. We therefore tested the models with spins rotating upon propagation through the crystal, which can potentially provide a constant moment solution. The only centrosymmetric model has a −1.1′ point group and implies that the spins on the Gd1_1 and Gd1_2 sites (as well as on Gd2_1 and Gd2_2) rotate in opposite ways within a given spin plane, which is not constrained by symmetry and can be absolutely general. This type of magnetic order averages to zero the magnetic interaction between the Gd1_1 and Gd1_2 (Gd2_1 and Gd2_2) sites and is therefore unlikely. The model also did not work in the refinement procedure and could therefore be safely ruled out. Any constant moment solution with the symmetry-related spins rotating in the same way upon propagation through the crystal, such as helix or cycloid (or admixture of both), necessarily implies broken spatial inversion and a polar point group. This is not always the case and in the present system, this is due to the low symmetry of the **k**_Gd_ propagation vector (plane of symmetry rather than a line of symmetry).

**Figure 5:**
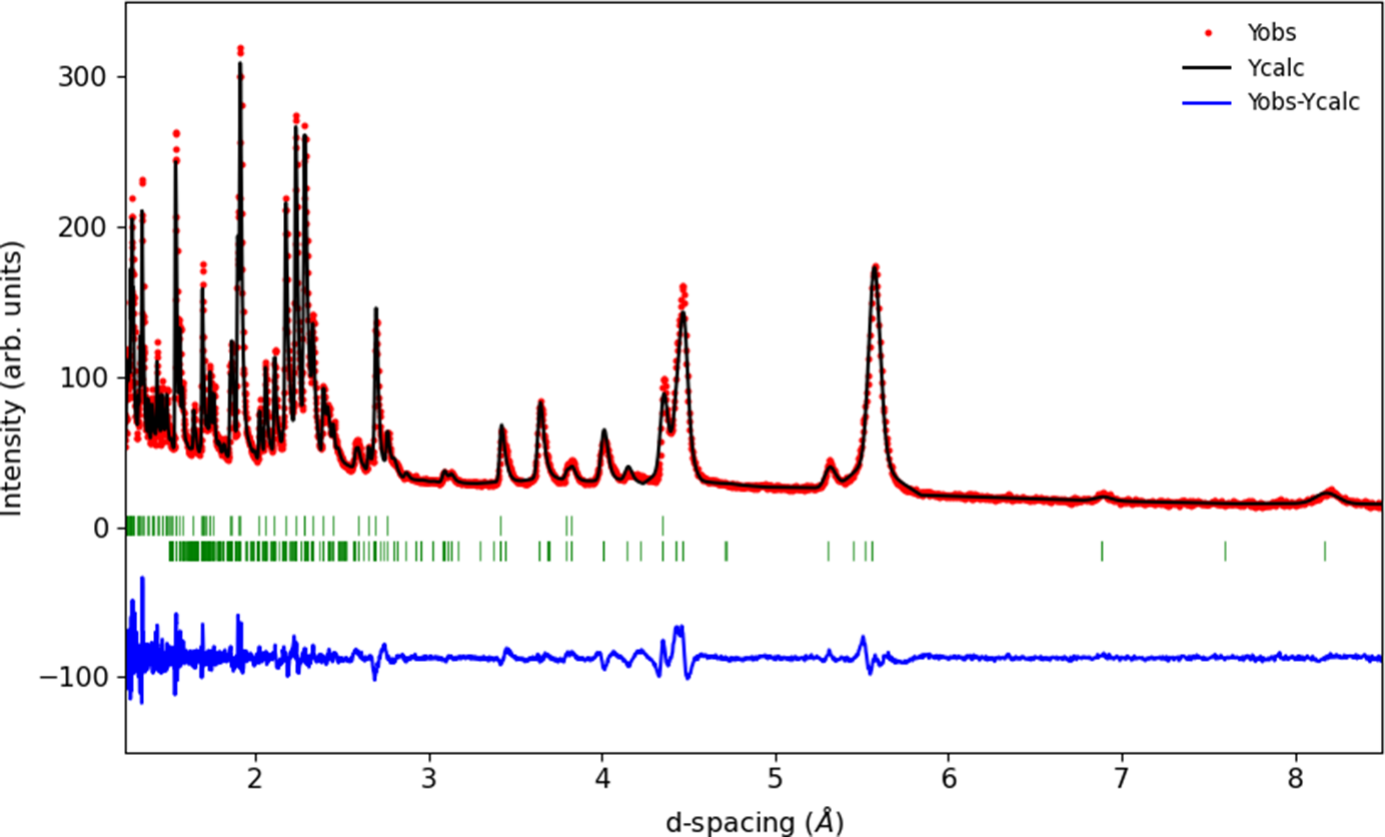
Rietveld refinement plot of the low-temperature phase of GdCrO_3_ at 1.5 K showing the observed data (red points) as well as the calculated (black line) and difference (blue lines) profiles. The green tick marks represent the positions of the nuclear (top) and magnetic (bottom) reflections.

One can distinguish two high-symmetry polar cases, with magnetic point groups *m*.1′ and 2.1′. In the former case, the spins are confined to be within the *ac* plane (cyclo­idal structure), while in the latter case, the plane of the spin rotations contains the *b* axis and intersects the *ac* plane along any general direction (admixture of helix and cycloid). Keeping the Gd1_1 and Gd1_2 (Gd2_1 and Gd2_2) spins antiferromagnetically coupled and refining the relative magnetic phase between the symmetry-unrelated Gd sites, we found that both models provide a good fit for the experimentally measured intensities. The model with the 2.1′ point group gives slightly better 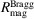 = 6.02% (versus 6.20% for the *m*.1′) making this magnetic structure the preferred candidate for the ground state of the Gd sublattice. This is also in agreement with the polarization observed along the *b* axis of *Pnma*.1′ and presented in Fig. 1 ▸. The resulting refinement is presented in Fig. 5 ▸. We also found that a significant improvement in the fitting quality can be achieved, by departing from the constant moment (circular envelope) structure towards a model with elliptical envelope. This would indicate the presence of significant magnetic anisotropy whose origin is not obvious for the isotropic Gd spins. The Fourier coefficients of the magnetic structure refined in both models are listed in Table 1 ▸. Due to the limited number of magnetic reflections in the powder diffraction data, we constrained the moment size to be the same for the symmetry-unrelated Gd sites.

### 3.5. Superspace group approach

Instead of the extended little group approach, the symmetry analysis could be performed using the superspace group formalism implemented in *ISODISTORT* software (Stokes *et al.*, 1995 ▸; Campbell *et al.*, 2006 ▸). The program indicates that there are two four-dimensional irreducible representations, mM1 and mM2 associated with **k**_Gd_ propagation vector. By restricting the incommensurate modulation down to a single arm of the wavevector star, one can find two nonpolar superspace groups, *P*2_1_/*m*.1′(α,β,0)00*s*, and *P*2_1_/*m*.1′(α,β,0)0*ss*. They correspond to the high-symmetry-order parameter direction (*a*,0;0,0) in mM1 and mM2, respectively. These structures represent a spin density wave either along the unique axis (mM1 irrep) or in the plane perpendicular to the unique axis (mM2 irrep). The general direction of the order parameter (*a*,*b*;0,0) for both irreps yields polar symmetry *Pm*.1′(α,β,0)0*s* and *Pm*.1′(α,β,0)*ss*. The former is a collinear acentric spin density wave while the latter implies the cyclo­idal order obtained in our refinement with the spins confined with the *ac* plane (Table 1 ▸). By combining both mM1 and mM2, one can get three distinct superspace groups, *P*2_1_.1′(α,β,0)0*s*, *P*1.1′(α,β,γ)0*s* and *P*1.1′(α,β,γ)0*s*. The first one represents the main candidate for the ground state magnetic order of the Gd-sublattice summarized in Table 1 ▸. We note that from a simple inspection of the MSG symbol, the point-group symmetry, relevant for the physical properties, can be readily obtained which is one of the advantages of this approach. The final refined structure, including the Cr moments can be described in MSSG *P*1(α,β,γ)*s* using the following transformation: *c*,*a*,*b*;0,0,0 (details given in Table 4 ▸). An .mcif file of the refined structure is provided as supporting information.

## 4. Discussion and conclusions

Whilst the high-temperature magnetic phase, emerging below 170 K, behaved as expected with refinements consistent with the mΓ_4_^+^ irrep, (MSG *Pn*′*ma*′), our detailed analysis using high-resolution neutron diffraction and symmetry analysis reveals a more complex behaviour than predicted. Not only is the spin re-orientation from mΓ_4_^+^ to mΓ_2_^+^ (MSG *P*2_1_′/*c*′) never fully completed but further influence of the Cr sublattice on the Gd sublattice can be clearly seen below 8 K through the appearance of weak and well resolved peaks only compatible with Gd ordering. Furthermore, an extra mode, characterized by the appearance of the 010 Bragg peak, is not included in these two irreps, implying an even more complicated spin re-orientation process, apparently via a triclinic phase. In contrast to a previous low-resolution study (Tripathi *et al.*, 2019 ▸), we do not see any evidence for a 010 reflection but the contribution of this reflection could be easily included in the background of the previous study.

Spin re-orientation phase transitions have been observed in many orthochromate and orthoferrite perovskites. This type of phase transition is usually mediated by *f*–*d* exchange and anisotropy of the rare earth sublattice and it is, therefore, rather surprising to observe it in a system with nominally isotropic Gd^3+^ spins. The fact that Gd^3+^ has no single ion anisotropy and Cr^3+^ has negligible anisotropy makes GdCrO_3_ a rather unique system within the orthochromate and orthoferrite families. Indeed, if the single ion terms are negligible, interactions such as Dzyaloshinskii–Moriya (DM) can become the next dominant term in the Hamiltonian. In GdCrO_3_, the oxygen octahedral tilting pattern is complex and their magnitude rather strong. The direction of Cr moments in the high-temperature phase (above the spin reorientation) is fully consistent with the dominant role of the DM interactions imposed by the in-phase and out-of-phase octahedral tilting (Khalyavin *et al.*, 2015 ▸). These distortions promote easy plane anisotropies, which intersect along the *c* axis. Analysis of the DM anisotropy associated with the *f*–*d* interaction is less straightforward and this anisotropy may differ from the *d*–*d* exchange, resulting in the observed spin reorientation,

The low-temperature phase appearing at 2.3 K and refined at *T* = 1.5 K [Gd only MSSG *P*2_1_.1′(α,β,0)0*s*] is also more complex than that generally observed in other orthochromates or orthoferrites for which it is unusual to observe an incommensurate phase, the exception being DyFeO_3_ which still boasts a simpler incommensurate structure that we observe for GdCrO_3_ with a **k** vector along a line of symmetry rather than a plane of symmetry.

We are now able to discuss the symmetry of the system taking into account both the Cr and Gd sublattices in the low-temperature phase. In the most symmetric case, when the ordering of the Cr sublattice is described by the single mΓ_2_^+^ irrep, the polar properties of the system are fully determined by the Gd sublattice as discussed above. The only difference is that the corresponding magnetic point group does not contain time reversal 1′ as a separate symmetry element. This mainly affects the magnetoelectric, but not the polar properties of the material. The most symmetric variants of the magnetic structures discussed above are described by the 2′/*m*′, *m*′ and 2′ points groups. The latter two represent symmetries of the system when the Gd sublattice adopts the ordering described in Table 1 ▸. If the Cr sublattice admixes both mΓ_2_^+^ and mΓ_4_^+^ irreps as in the model used to refine the 1.5 K data (see Table 1 ▸), then the two solutions with *m*′ and 2′ points groups do not represent symmetry distinct cases, and both are characterized by the same point group 1 with clear consequences for the possible direction of the polarisation. This case also further relaxes the direction of the Cr moments allowing admixture of additional irreps, including mΓ_1_^+^.

Finally, we note that the solution obtained has an elliptical envelope. Our permittivity data show a further transition at ∼1.4 K, which we could speculate to be related to the envelope becoming circular, more appropriate for the ground state. This transition would be isostructural as the symmetry is already *P*1 which would be consistent with the presence of a hysteresis in the permittivity. At these temperatures, the influence of dipolar interactions between the large Gd moments would also likely play a role.

## Supplementary Material

Supporting information file. DOI: 10.1107/S2052520625001921/gar5006sup1.mcif

## Figures and Tables

**Table 1:** Magnetic moments on each Cr and Gd site in Bohr magnetons refined at different temperatures 20 K (HT phase, mΓ_4_^+^, MSG *Pn*′*ma*′), 5 K (incomplete spin reorientation phase mΓ_4_^+^ to mΓ_2_^+^, MSG *P*2_1_′/*c*′) and 1.5 K [Gd ordering incommensurate MSSG *P*2_1_.1′(α,β,0)0*s*] Atomic coordinates of the Cr and Gd sites are: Cr1(0, 0, ½), Cr2(0, ½,−½), Cr3 (½, 0, 1), Cr4 (½, ½, 0), Gd11 (0.05888, ¼, −0.01540), Gd12 (−0.05888, 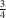, 0.01540), Gd21 (0.44112, 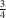, 0.48460), Gd22 (0.55888, ¼, 0.51540). 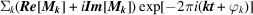 + 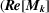 − 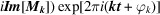, where 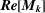 and 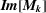 are real and imaginary parts of the Fourier coefficients, defining the spin plane, **t** is a lattice translation and φ_*k*_ is a magnetic phase.

		Cr1	Cr2	Cr3	Cr4	Gd1_1	Gd1_2	Gd2_1	Gd2_2
*T* = 20 K									
**k**_Cr_	*M*_*x*_	–	–	–	–	–	–	–	–
	*M*_*y*_	–	–	–	–	–	–	–	–
	*M*_*z*_	2.36 (2)	−2.36 (2)	−2.36 (2)	2.36 (2)	–	–	–	–

*T* = 5 K									
**k**_Cr_	*M*_*x*_	–	–	–	–	0.53 (4)	0.53 (4)	−0.53 (4)	−0.53 (4)
	*M*_*y*_	2.06 (3)	−2.06 (3)	−2.06 (3)	2.06 (3)	–	–	–	–
	*M*_*z*_	1.13 (5)	−1.13 (5)	−1.13 (5)	1.13 (5)	–	–	–	–

*T* = 1.5 K									
**k**_Cr_	*M*_*x*_	–	–	–	–	1.07 (4)	1.07 (4)	−1.07 (4)	−1.07 (4)
	*M*_*y*_	1.92 (7)	−1.92 (7)	−1.92 (7)	1.92 (7)	–	–	–	–
	*M*_*z*_	1.65 (6)	−1.65 (6)	−1.65 (6)	1.65 (6)	–	–	–	–

Magnetic point group 2.1′									
**k**_Gd_									
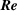	*M*_*x*_	–	–	–		–	–	–	–
	*M*_*y*_	–	–	–		–	–	–	–
	*M*_*z*_	–	–	–		4.70 (8)	4.70 (8)	4.70 (8)	4.70 (8)
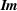	*M*_*x*_	–	–	–		–	–	–	–
	*M*_*y*_	–	–	–		2.2 (1)	2.2 (1)	2.2 (1)	2.2 (1)
	*M*_*z*_	–	–	–		–	–	–	–
	φ_*k*_	–	–	–		0	0.5	0.244 (6)	0.744 (6)

Magnetic point group *m*.1′									
**k**_Gd_									
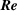	*M*_*x*_	–	–	–		–	–	–	–
	*M*_*y*_	–	–	–		–	–	–	–
	*M*_*z*_	–	–	–		4.76 (7)	4.76 (7)	4.76 (7)	4.76 (7)
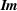	*M*_*x*_	–	–	–					
	*M*_*y*_	–	–	–		2.22 (8)	2.22 (8)	2.22 (8)	2.22 (8)
	*M*_*z*_	–	–	–		–	–	–	–
	φ_*k*_	–	–	–		0	0.5	0.234 (8)	0.734 (8)

**Table 2:** Crystallographic details of the *Pn*′*ma*′ phase obtained at 20 K

Parent space group	*Pnma*.1′
Propagation vector(s)	(0,0,0)
Transformation from parent basis to the one used	*a*,*b*,*c*; 0,0,0
MSG symbol	*Pn*′*ma*′
MSG number	62.448
Transformation from basis used to standard setting of MSG	*a*,*b*,*c*; 0,0,0
Magnetic point group	*m*′*m*′*m*
Active irreps	mΓ_4_^+^, special direction, primary-order parameter
Unit-cell parameters (Å, °)	*a* = 5.52013 (9), *b* = 7.59296 (13), *c* = 5.30454 (9), α = 90.0, β = 90.0, γ = 90.0
MSG symmetry operations (8)	*x*, *y*, *z*, +1
	−*x*, *y* + ½, −*z*, +1
	−*x*, −*y*, −*z*, +1
	*x*, −*y* + ½, *z*, +1
	*x* + ½, −*y* + ½, −*z* + ½, −1
	−*x* + ½, −*y*, *z* + ½, −1
	−*x* + ½, *y* + ½, *z* + ½, −1
	*x* + ½, *y*, −*z* + ½, −1
Positions of magnetic atoms	Gd1 0.05879 0.25000 −0.01500
	Cr1 0.00000 0.00000 0.50000
Positions of nonmagnetic atoms	O11 0.4740 0.25000 0.0947
	O21 0.2977 0.0498 0.6990
Magnetic moment components (μ_B_) of magnetic atoms, symmetry constraints and moment magnitude	Cr1 0.0 0.0 0.0, *M*_*x*_, *M*_*y*_, *M*_*z*_, 0.0
	Cr1 0.0 0.0 2.359 (18), *M*_*x*_, *M*_*y*_, *M*_*z*_, 2.359 (18)

**Table 3:** Crystallographic details of the *Pmma*.1′ phase obtained at 5 K

Parent space group	*Pnma*.1′
Propagation vector(s)	(0,0,0)
Transformation from parent basis to the one used	−*c*, −*a*, *b*+*c*; 0,0,0
MSG symbol	*P*2_1_′/*c*′
MSG number	14.79
Transformation from basis used to standard setting of MSG	*a*, *b*, *c*; 0,0,0
Magnetic point group	2′/*m*′
Active irreps	mΓ_4_^+^, special direction, primary-order parameter
	mΓ_2_^+^, special direction, primary-order parameter
Unit-cell parameters (Å, °)	*a* = 5.30453, *b* = 5.52033, *c* = 9.26231, α = 90.0, β = 124.93874, γ = 90.0
MSG symmetry operations (4)	*x*, *y*, *z*, +1
	−*x*, −*y*, −*z*, +1
	−*x*, *y* + ½, −*z* + ½, −1
	*x*, −*y* + ½, *z* + ½, −1
Positions of magnetic atoms	Gd1_1 Gd 0.26500 −0.05879 0.25000
	Cr1_1 Cr 0.50000 0.00000 0.00000
	Cr1_2 Cr 0.00000 0.00000 0.50000
Positions of nonmagnetic atoms	O1_1 O 0.15440 0.52610 0.25000
	O2_1 O 0.35080 0.70200 0.04990
	O2_2 O 0.24900 0.29800 0.54990
Magnetic moment components (μ_B_) of magnetic atoms, symmetry constraints and moment magnitudes	Gd1_1 0.0 −0.53 (4) 0.0, *M_x_*, *M_y_*, *M_z_*, −0.53 (4)
	Cr1_1 0.309 (13) 0.0 2.51 (4); *M_x_*, *M_y_*, *M_z_*, 2.35 (4)
	Cr1_2 −0.309 (13) 0.0 −2.51 (4); *M_x_*, *M_y_*, *M_z_*, 2.35 (4)

**Table 4:** Crystallographic details of the incommensurate *P*1(*abg*)*s* phase obtained at 1.5 K The modulation function is specified as: 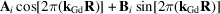, *i* = *x*, *y*, *z*, **R** = (**r** + **t**), **r** is atomic position in a cell, **t** is lattice translation.

Parent space group	*Pnma*
Propagation vector(s)	(0.3294, 0, 0.080)
Transformation from parent basis to the one used	*c*,*a*,*b*; 0,0,0
MSSG symbol	*P*1(*abg*)*s*
Transformation from basis used to standard setting of MSG	*a*,*b*,*c*; 0,0,0
Magnetic point group	1
Active irreps	mΓ_4_^+^, special direction, primary-order parameter
	mΓ_2_^+^, special direction, primary-order parameter
	m*M*_1_, special direction, primary-order parameter
	m*M*_2_, special direction, primary-order parameter
Unit-cell parameters (Å, °)	*a* = 5.30453, *b* = 5.52033, *c* = 7.5929, α = 90.0, β = 90.0, γ = 90.0
MSG symmetry operations (1S)	*x*1, *x*2, *x*3, *x*4, +1
Positions of magnetic atoms (**r**)	Gd1_1 −0.015 0.05879 0.25
	Gd1_2 0.015 −0.05879 0.75
	Gd1_3 0.515 0.55879 0.25
	Gd1_4 −0.515 −0.55879 0.75
	Cr1_1 0.5 0 0
	Cr1_2 −0.5 0 0.5
	Cr1_3 0 0.5 0.5
	Cr1_4 0 −0.5 1
Positions of nonmagnetic atoms	O1_1 O 0.0956 0.4739 0.25
	O1_2 O −0.0956 −0.4739 0.75
	O1_3 O 0.4044 −0.0261 0.25
	O1_4 O −0.4044 0.0261 0.75
	O2_1 O 0.6991 0.298 0.0499
	O2_2 O −0.6991 −0.298 0.5499
	O2_3 O 0.8009 0.798 0.4501
	O2_4 O −0.8009 −0.798 0.9501
	O2_5 O 0.3009 0.702 −0.0499
	O2_6 O −0.3009 −0.702 0.4501
	O2_7 O 0.1991 0.202 0.5499
	O2_8 O −0.1991 −0.202 1.0499
Magnetic moment components (μ_B_) of magnetic atoms, symmetry constraints and moment magnitudes	Gd1_1 0 1.07 0, *M_x_*, *M_y_*, *M_z_*, 1.07
	Gd1_2 0 1.07 0, *M_x_*, *M_y_*, *M_z_*, 1.07
	Gd1_3 0 −1.07 0; *M_x_*, *M_y_*, *M_z_*, 1.07
	Gd1_4 0 −1.07 0, *M_x_*, *M_y_*, *M_z_*, 1.07
	Cr1_1 1.641 0 1.92, *M_x_*, *M_y_*, *M_z_*, 2.35
	Cr1_2 −1.64 0 −1.92, *M_x_*, *M_y_*, *M_z_*, 2.35
	Cr1_3 1.64 0 1.94, *M_x_*, *M_y_*, *M_z_*, 2.35
	Cr1_4 −1.64 0 −1.92, *M_x_*, *M_y_*, *M_z_*, 2.35
Fourier component of the incommensurate modulation [atom, component (*i* = *x*,*y*,*z*), cosine (**A**_*i*_), sine (**B**_*i*_)]	Gd1_1 *x* 0.0 4.7
	Gd1_1 *y* 0.0, 0.0
	Gd1_1 *z* 2.2 0.0
	Gd1_2 *x* 0.0 4.7
	Gd1_2 *y* 0.0 0.0
	Gd1_2 *z* 2.2 0.0
	Gd1_3 *x* −4.7 0.0
	Gd1_3 *y* 0.0 0.0
	Gd1_3 *z* 0.0 −2.2
	Gd1_4 *x* 4.7 0.0
	Gd1_4 *y* 0.0 0.0
	Gd1_4 *z* 0.0 2.2
	Cr1_1 *x* 0.0 0.0
	Cr1_1 *y* 0.0 0.0
	Cr1_1 *z* 0.0 0.0
	Cr1_2 *x* 0.0 0.0
	Cr1_2 *y* 0.0 0.0
	Cr1_2 *z* 0.0 0.0
	Cr1_3 *x* 0.0 0.0
	Cr1_3 *y* 0.0 0.0
	Cr1_3 *z* 0.0 0.0
	Cr1_4 *x* 0.0 0.0
	Cr1_4 *y* 0.0 0.0
	Cr1_4 *z* 0.0 0.0

## References

[bb1] Belov, N. V., Neronova, K. N. & Smirnova, T. S. (1957). *Sov. Phys. Crystallogr.* **2**, 311–322.

[bb2] Bertaut, E. F. (1963). In *Magnetism*, Vol, 3, edited by G. T. Rado and H. Suhl. Academic Press.

[bb3] Campbell, B. J., Stokes, H. T., Tanner, D. E. & Hatch, D. M. (2006). *J. Appl. Cryst.* **39**, 607–614.

[bb4] Chapon, L. C., Manuel, P., Radaelli, P. G., Benson, C., Perrott, L., Ansell, S., Rhodes, N. J., Raspino, D., Duxbury, D., Spill, E. & Norris, J. (2011). *Neutron News*, **22**, 22–25.

[bb5] Cheng, Z. X., Wang, X. L., Dou, S. X., Kimura, H. & Ozawa, K (2010). *J. Appl. Phys.* **107**, 09D905.

[bb8] Cooke, A. H., Martin, D. M. & Wells, M. R. (1974). *J. Phys. C Solid State Phys.* **7**, 3133–3144.

[bb9] Geller, S. (1957). *Acta Cryst.* **10**, 243–248.

[bb10] Khalyavin, D. D., Salak, A. N., Manuel, P., Olekhnovich, N. M., Pushkarev, A. V., Radysh, Y. V., Fedorchenko, A. V., Fertman, E. L., Desnenko, V. A. & Ferreira, M. G. S. (2015). *Z. Kristallogr. Crystall. Mater.* **230**, 767–774.

[bb11] Kimura, T., Goto, T., Shintani, H., Ishizaka, K., Arima, T. & Tokura, Y. (2003). *Nature*, **426**, 55–58. 10.1038/nature02018 14603314

[bb12] Mareschal, J. & Sivardière, J. (1969). *J. Phys. Paris*, **30**, 967.

[bb13] Perez-Mato, J. M., Gallego, S. V., Tasci, E. S., Elcoro, L., de la Flor, G. & Aroyo, M. I. (2015). *Annu. Rev. Mater. Res.* **45**, 217–248.

[bb14] Ramesha, K., Llobet, A., Proffen, T., Serrao, C. R. & Rao, C. N. R. (2007). *J. Phys. Condens. Matter*, **19**, 102202.

[bb15] Ritter, C., Vilarinho, R., Moreira, J. A., Mihalik, M., Mihalik, M. & Savvin, S. (2021). *J. Phys. Condens. Matter*, **34**, 265801. 10.1088/1361-648X/ac6787 35421851

[bb16] Serrao, C. R., Kundu, A. K., Krupanidhi, S. B., Waghmare, U. V. & Rao, C. N. R. (2005). *Phys. Rev. B*, **72**, 220101.

[bb17] Spaldin, N. A. & Fiebig, M. (2005). *Science*, **309**, 391–392. 10.1126/science.1113357 16020720

[bb6] Stokes, H. T., Hatch, D. M. & Campbell, B. J. (1995). *ISODISTORT*, *ISOTROPY Software Suite*. https://stokes.byu.edu/iso/isotropy.php

[bb18] Tripathi, M., Chatterji, T., Fischer, H. E., Raghunathan, R., Majumder, S., Choudhary, R. J. & Phase, D. M. (2019). *Phys. Rev. B*, **99**, 014422.

[bb19] Wanklyn, B. M. (1969). *J. Cryst. Growth*, **5**, 323–328.

[bb20] Yin, L. H., Yang, J., Kan, X. C., Song, W. H., Dai, J. M. & Sun, Y. P. (2015). *J. Appl. Phys.* **117**, 133901.

